# Oct4 promotes M2 macrophage polarization through upregulation of macrophage colony-stimulating factor in lung cancer

**DOI:** 10.1186/s13045-020-00887-1

**Published:** 2020-06-01

**Authors:** Chia-Sing Lu, Ai-Li Shiau, Bing-Hua Su, Tsui-Shan Hsu, Chung-Teng Wang, Yu-Chu Su, Ming-Shian Tsai, Yin-Hsun Feng, Yau-Lin Tseng, Yi-Ting Yen, Chao-Liang Wu, Gia-Shing Shieh

**Affiliations:** 1 Department of Biochemistry and Molecular Biology, College of Medicine, National Cheng Kung University, 1, University Road, Tainan, Taiwan; 2 Department of Microbiology and Immunology, College of Medicine, National Cheng Kung University, Tainan, Taiwan; 3 School of Respiratory Therapy, College of Medicine, Taipei Medical University, Taipei, Taiwan; 4 Division of Thoracic Surgery, Department of Surgery, College of Medicine, National Cheng Kung University, Tainan, Taiwan; 5 Division of Hematology and Oncology, Department of Internal Medicine, Chi-Mei Medical Center, Tainan, Taiwan; 6 Department of Urology, Tainan Hospital, Ministry of Health and Welfare, Executive Yuan, Tainan, Taiwan

**Keywords:** Lung cancer, Oct4, M2 macrophage, Tumor-associated macrophage, M-CSF

## Abstract

**Background:**

Expression of Oct4 maintains cancer stem cell (CSC)-like properties in lung cancer cells and is correlated with poor prognosis of lung adenocarcinoma. M2-type tumor-associated macrophages (TAMs) promote cancer cell migration and metastasis. Tumor microenvironments promote monocyte differentiation into M2 TAMs via a complex cytokine-based connection. We explored the role of Oct4 in cytokine secretion in lung cancer and its impact on M2 TAM polarization.

**Methods:**

Monocytes co-cultured with the conditioned medium from Oct4-overexpressing lung cancer cells were used to investigate M2 TAM differentiation. The inflammatory factors in the conditioned medium of Oct4-overexpressing A549 cells were examined using human inflammation antibody arrays. The correlations of Oct4, macrophage colony-stimulating factor (M-CSF), and M2 TAMs were validated in lung cancer cells, syngeneic mouse lung tumor models, and clinical samples of non-small cell lung cancer (NSCLC).

**Results:**

Oct4-overexpressing A549 cells expressed elevated levels of M-CSF, which contributed to increased M2 macrophages and enhanced tumor migration. Overexpression of Oct4 enhanced tumor growth and reduced the survival of lung tumor-bearing mice, which was correlated with increased number of M2 macrophages in lung cancer. Notably, NSCLC patients with high expression levels of Oct4, M-CSF, and M2 TAMs had the poorest recurrence-free survival. A positive correlation between Oct4, M-CSF, and M2 TAMs was observed in the tumor tissue of NSCLC patient. Treatment with all-trans retinoic acid exerted anti-tumor effects and reduced M2 TAMs in tumor-bearing mice.

**Conclusions:**

Our results indicate that Oct4 expressed by lung cancer cells promotes M2 macrophage polarization through upregulation of M-CSF secretion, leading to cancer growth and metastasis. Our findings also implicate that the Oct4/M-CSF axis in M2 macrophage polarization may be potential therapeutic targets for lung cancer.

## Background

Lung cancer is the leading cause of cancer-related mortality worldwide. Immune cells can interact with tumor cells and change tumor phenotypes that may actually contribute to the establishment of a tumor-supporting environment [1]. It was shown that the numbers of tumor-associated immune cells were significantly greater in stage III than in stage I lung cancer specimens [2]. The roles of tumor-associated macrophages (TAMs) in tumor progression and metastasis have gained much attention [3]. Monocytes are recruited into hypoxic regions of tumors and other ischemic tissues by chemoattractants [4]. Several studies have shown that monocytes can be recruited to tumor sites through the secretion of various mediators, such as macrophage colony-stimulating factor (M-CSF), monocyte chemoattractant protein (MCP)-1, MCP-2, macrophage inflammatory protein (MIP)-1α, and MIP-1β [5]. In response to microenvironmental signals, macrophages can undergo two forms of activations. M1 macrophages are anti-tumorigenic, whereas M2 macrophages are pro-tumorigenic. The Th2 cytokines produced by M2 macrophages act in favor of tumor progression in lung adenocarcinoma [6] and epithelial-mesenchymal transition (EMT) in pancreatic cancer cells [7]. The M1/M2 gene signatures have been identified and correlated with the survival of non-small cell lung cancer (NSCLC) patients [8]. Identification and quantification of these immune cell populations and their roles in lung cancer are currently being investigated.

M-CSF is the primary growth factor that regulates the growth, proliferation, and differentiation of cells of hematopoietic lineages, including monoblasts, promonocytes, monocytes, macrophages, and osteoclasts [9, 10]. M-CSF is secreted by various types of cells, including monocytes, fibroblasts, osteoblasts, stromal cells, endothelial cells, and tumor cells. Previous studies have demonstrated an elevation of M-CSF in the sera of patients with different tumor types, including ovarian cancer and lung cancer [11, 12].

Expression of Oct4 maintains cancer stem cell (CSC)-like properties in lung cancer cells [13] and is correlated with poor prognosis of lung adenocarcinoma [14]. We have reported previously that Oct4 is detected and predicts bladder cancer progression and metastasis [15]. Recently, we also showed that Oct4 induced by chemotherapeutic drugs can enhance the acquisition of CSC phenotypes and drug resistance in bladder cancer, and that Oct expression is positively correlated with tumor recurrence [16]. Oct4 expression increases tumorigenic properties of colorectal cancer cells through the secretion of IL-8 and IL-32 [17]. IL-8 secreted by cancer cells can induce EMT and angiogenesis (autocrine effect), as well as recruitment of monocytes, neutrophils, and other leukocytes (paracrine effect) [18]. Collectively, these findings suggest that expression of Oct4 in cancer cells may influence TAMs through cytokine secretion.

Overexpression of Oct4 is correlated with poor prognosis in cancer patients [14–16]. However, the link between Oct4 expression in cancer cells and TAMs within the tumor microenvironments remains unclear. In the present study, we investigated how overexpression of Oct4 promoted lung cancer progression in the tumor microenvironment. Our results show that tumor necrosis factor-α (TNF-α) and IL-1β upregulated Oct4 expression in lung cancer cells, which may in turn transcriptionally activated M-CSF expression. The secreted M-CSF increased M2 macrophages within tumors and thereby promoted tumor metastasis. Additionally, we demonstrate positive correlations between the expression levels of Oct4 and those of M-CSF, as well as between the levels of Oct4 and those of M2 macrophages in clinical specimens of lung cancer. Importantly, high levels of Oct4, M-CSF, and CD206 in combination are correlated with poor prognosis of patients with lung cancer. Collectively, these novel findings support a central role for Oct4 and TAMs in lung tumor progression and reveal that Oct4 may be a potential therapeutic target for lung cancer.

## Methods

### Clinical specimens

Two cohorts of different hospitals were examined independently. For immunohistochemical staining of Oct4, M-CSF, and CD206, 22 specimens were collected from patients with lung adenocarcinoma at the Thoracic Division, Department of Surgery, National Cheng Kung University (NCKU) Hospital. Informed consent was obtained from all subjects, and the experimental protocol was approved by the Institutional Review Board (IRB), NCKU Hospital (IRB number: B-ER-105-406). Another cohort with a total of 84 patients diagnosed with lung cancer between September 1998 and March 2010 at the Department of Pathology of Chi-Mei Medical Center, Tainan, Taiwan, was included. This study was approved by the IRB of the Chi-Mei Medical Center (approval no. 10308-002). There were no restrictions on gender, histological subtype, or stage. Patients were surgically treated for primary lung cancer, including 68 adenocarcinomas, 14 squamous cell carcinomas, one adenosquamous carcinoma, and one atypical carcinoid tumor. The clinicopathological parameters of the patients are provided (Additional file 1: Table S1).

### Cells, mice, and recombinant proteins

Human NSCLC (A549, H1299, CL1-0, CL1-1, and CL1-5-F4), embryonic kidney 293 T, and monocytic THP-1, as well as murine lung adenocarcinoma LL2 (Lewis lung carcinoma) cell lines, were used in this study. A549, LL2, and THP-1 cells were purchased from the Bioresource Collection and Research Center (BCRC, Hsinchu, Taiwan). H1299 cells were obtained from J.-Y. Chen (Graduate Institute of Microbiology, College of Medicine, National Taiwan University) [19]. CL1-0, CL1-1, and CL1-5-F4 cell lines were kindly provided from P.-C. Yang (Department of Internal Medicine, National Taiwan University) [20]. Stable Oct4-overexpressing A549 (designated A549-Oct4) and vector control cells were established by transduction with lentiviruses expressing Oct4 and control lentiviruses, respectively, followed by puromycin selectin [16]. THP-1 cells were differentiated into macrophages by treatment with 320 nM of phorbol 12-myristate 13-acetate (PMA) for 6 h and then cultured for an additional 16 h with either PMA only to generate M0 cells or PMA plus lipopolysaccharide (100 ng/ml)/interferon-γ (20 ng/ml) to generate M1-polarized cells. All cells were cultured in Dulbecco’s modified Eagle’s medium (DMEM) supplemented with 10% cosmic calf serum (Hyclone, Logan, UT), 2 mM L-glutamine, and 50 μg/ml gentamicin at 37 °C in 5% CO_2,_ except that THP-1 cells were cultured in RPMI 1640 medium supplemented with 10% fetal bovine serum (FBS). Recombinant proteins used in this study were purchased from PeproTech (London, UK). Male 6- to 8-week-old C57BL/6 mice were obtained from the Laboratory Animal Center of NCKU. All animal experiments were conducted following the guidelines approved by the Institutional Animal Care and Use Committee of NCKU (approval no. 98023).

### Lentiviral and retroviral vectors

The lentiviral expressing vector encoding human Oct4 (pSin-EF2-Oct4-Pur, Addgene plasmid 16579) was obtained from Addgene (Cambridge, MA) [21]. The lentiviral control vector pSin-null was derived from pSin-EF2-Oct4-Pur as previously described [16]. For knockdown experiments, pLKO.1-puro-based lentiviral vectors, including stem-loop cassettes encoding shRNA for human Oct4 (TRCN0000004880 and TRCN0000004883, designated shOct4 TRCN4880 and shOct4 TRCN4883) and for luciferase (TRCN 0000072246, designated shLuc), were obtained from the National RNAi Core Facility, Academia Sinica, Taiwan. All recombinant lentiviruses were produced and titrated as previously described [16].

The retroviral expressing vector encoding mouse Oct4 (pMXs-Oct3/4, Addgene plasmid 13366) and retroviral empty backbone vector pBABE-puro (Addgene plasmid 1764) were obtained from Addgene [22, 23]. The RetroMax system (Imgenex, San Diego, CA) was used to produce recombinant retroviruses according to the manufacturer’s instructions.

### Luciferase reporter constructs

To assess the promoter activity of the *M-CSF* gene, we generated luciferase reporter constructs containing wild-type and mutant *M-CSF* promoters based on a single dual-luciferase reporter plasmid pFRL2 [24]. The *M-CSF* promoter region (from −1983 to +1 bp relative to the transcription start site) was obtained from the genomic DNA of 293 T cells using the polymerase chain reaction (PCR) with the primer pairs 5′-TACACAGCAAATGAATGGCAGAGCTGG-3′ (forward) and 5′-GCGTCTTCCTAGTCACCCTCTGTCTTCTG-3′ (reverse), and cloned into the TA cloning vector yT&A, excised by digestion with *Sma*I/*Bgl*II, and cloned into the *Sma*I/*Bgl*II sites of pFRL2 to generate pFRL2*-*M-CSF(−1983 ~ +1). We also constructed four deletion mutants in the *M-CSF* promoter region encompassing −1063 ~ +1, −903 ~ +1, −577 ~ +1, and −487 ~ +1 bp (from the transcription start site). The promoter regions of the four deletion mutants were obtained from pFRL2-M-CSF(−1983 ~ +1) by PCR with the forward primers, including (−1063) 5′-ACTGCACGCGTATGAGCCAAGTCCA-3′, (−903) 5′-TCTGCACGCGTCAGTCTGAGCAAAG-3′, (−577) 5′-CATGGACGCGTTTCCAATCTGAGTTG-3′, and (−487) 5′-TAAGGACGCGTTGAAGTGTCTGCTGG-3′, as well as the reverse primer 5′-TATATCTCGAGCACCCTCTGTCTTCTGCG-3′. The PCR products were then ligated into the yT&A vector. These promoter regions with various deletions were then excised from the TA vector by digestion with *Xho*I/*Mlu*I (underlined) and cloned into the *Xho*I/*Mlu*I sites of pFRL2 to generate pFRL2-M-CSF(−1063 ~ +1), pFRL2-M-CSF(−903 ~ +1), pFRL2-M-CSF(−577 ~ +1), and pFRL2-M-CSF(−487 ~ +1). In addition, three luciferase reporter vectors containing the *M-CSF* promoter carrying a point mutation (ATGCAATT ➔ ACGCGATT) at −980 bp within the first Oct4 response element (ORE1) site, a point mutation (ATGCAAAT ➔ CTGAAGAT) at −530 bp within the second ORE site (ORE2), and a double mutation within both ORE1 and ORE2 sites were generated using pFRL2-M-CSF(−1983 ~ +1) as the template by site-directed mutagenesis by overlap extension using PCR [25]. The primers (point mutation underlined) used include mutant 1, 5′-GAGACGCGATTTCAGCCTGAAATGATGAGGAGTT-3′ (forward) and 5′-CTGAAATCGCGTCTCATCCTCCACCAGCAAAGC-3′ (reverse); mutant 2, 5′-GCATCTTCAGCATCTAAGGGTCAGGTGCCTTGAA-3′ (forward) and 5′-TGCTGAAGATGCTGGCTGGTACCCATGCT-3′ (reverse); and pFRL2, 5′-CCAGCCCAAGCTACCATGATAAGTAAG-3′ (forward) and 5′-CTTATGCAGTTGCTCTCCAGCGG-3′ (reverse). Finally, two outer primers 5′-TACACAGCAAATGAATGGCAGAGCTGG-3′ (forward) and 5′-GCGTCTTCCTAGTCACCCTCTGTCTTCTG-3′ (reverse) were used to synthesize the entire DNA sequence by PCR. The PCR products were cloned into the yT&A vector. The resulting constructs were then digested with *Eco*RI/*Bgl*II, and the released mutant fragments were cloned into the *Eco*RI/*Bgl*II sites of pFRL2 to generate pFRL2-M-CSF-ORE1mut (designated Mut1), pFRL2-M-CSF-ORE2mut (designated Mut2), and pFRL2-M-CSF-ORE1mut-ORE2mut (designated Dmut). All the cloned fragments were sequenced to validate their correctness.

### Reverse transcription-PCR (RT-PCR)

Total RNA (2 μg) was reverse transcribed into cDNA using the Verso™ cDNA synthesis kit (Thermo Fisher Scientific, San Jose, CA). The primers used include human Oct4, 5′-GTCCGAGTGTGGTTCTGTA-3′ (forward) and 5′-CTCAGTTTGAATGCATGGGA-3′ (reverse); human M-CSF, 5′-CTAAGCTGGACGCACAG ACCA-3′ (forward) and 5′-TCTCAGGCTGCACACCTT-3′ (reverse); human glyceraldehyde-3-phosphate dehydrogenase (GAPDH), 5′-GCCATCACTGCCACCC AG-3′ (forward) and 5′-TCTTACTCCTTGGAGGCCATGT-3′ (reverse). The amplicons of Oct4, M-CSF, and GAPDH were 264, 538, and 496 bp, respectively.

### Luciferase reporter assay

Subconfluent 293 T or H1299 cells were cotransfected with various amounts of pSin-EF2-Oct4-Pur (or control vector pSin-null) and pFRL2-M-CSF (or mutant luciferase reporter plasmid) using Polyethylenimine 25,000 (0.45 mg/ml, pH 7.0). Cell lysates were harvested 48 h after transfection, and their firefly and *Renilla* luciferase activities were determined using a dual-light luciferase reporter assay system (Promega, Madison, WI). The ratio of firefly luciferase activity to *Renilla* luciferase activity was expressed as relative light units (RLU).

### Chromatin immunoprecipitation (ChIP) assay

ChIP was performed as previously described [26]. Genomic DNA of A549-Oct4 or H1299 cells that had been sheared by sonication to an average length of 500 bp were immunoprecipitated with mouse anti-Oct4 antibody (C-10, sc-5279, Santa Cruz Biotechnology, Santa Cruz, CA) or normal mouse IgG2a (sc-3878, Santa Cruz) in combination with protein G agarose beads. The primers used for PCR analysis of immunopurified DNA and input samples include 5′-GTACTGTGGAGAATGAATGGATGGCAA′-3′ (forward) and 5′-ATGATGAGGAGTTTGTCTTCAGCCATG-3′ (reverse) corresponding to the ORE1 site of the *M-CSF* promoter, as well as 5′-CATCCAGGGAAATCTAGGGTCCAGG-3′ (forward) and 5′-GTCAGGTGCCTTGAAGTGTCTGCTG-3′ (reverse) corresponding to the ORE2 fragment of the *M-CSF* promoter.

### Flow cytometric analysis

To analyze macrophages infiltrating into tumors, tumor-bearing mice were sacrificed at different time points, and tumor mass was incubated with collagenase (100 μg/ml) at 37 °C for 30 min. The isolated tumor cells (1 × 10^6^) were filtered through a mesh, and washed twice and resuspended in FACS buffer (PBS containing 1% FBS and 0.1% sodium azide). Cells were then stained with PE-Cy7-conjugated rat anti-mouse F4/80 (25-4801-82, eBioscience), FITC-conjugated rat anti-mouse CD86 (553691, BD Biosciences), or FITC-conjugated rat anti-mouse CD206 (141704, BioLegend, San Diego, CA) antibody. In addition, PMA-stimulated THP-1 cells that had been cocultured with A549-Oct4 or vector control cells were stained with PE-conjugated mouse anti-human CD86 (560957, BD Biosciences) or CD206 (555954, BD Biosciences) antibody for 30 min in 100 μl of FACS buffer. Cells were then fixed and permeabilized with a fixation and permeabilization solution (554722, BD Biosciences). After washing with BD Perm/Wash buffer (554723, BD Biosciences), the cells were stained with FITC-conjugated anti-human CD68 antibody (562117, BD Biosciences). After 30 min, the cells were washed three times with PBS and resuspended in 1 ml of FACS buffer for flow cytometric analysis.

### Immunohistochemistry, immunoblotting, and enzyme-linked immunosorbent assay (ELISA)

Immunohistochemical staining was performed on formalin-fixed, paraffin-embedded sections of human and mouse lung tumor tissues as previously described [16]. Primary antibodies include rabbit polyclonal anti-human Oct4 (2750, Cell Signaling, Beverly, MA), rabbit monoclonal anti-human M-CSF (ab52864, Abcam, Cambridge, MA), and rabbit polyclonal anti-human/mouse CD206 (ab64693, Abcam). The immunoreactive intensity was analyzed with the HistoQuest analysis software (TissueGnostics, Vienna, Austria). Immunoblot analysis was performed to detect Oct4 and β-actin (as the loading control) using rabbit polyclonal anti-human Oct4 antibody (2750, Cell Signaling) and mouse monoclonal anti-β-actin-peroxidase antibody (A3854, Sigma-Aldrich, St. Louis, MO), respectively. For ELISA, A549 cells (1 × 10^6^) that had been transduced with recombinant lentiviruses derived from pSin-null, pSin-EF2-Oct4, shLuc, shOct4#4880, or shOct4#4883 lentiviral vectors were cultured in 6-well plates, and their conditioned media were collected after 72 h for quantifying the levels of M-CSF, TNF-α, IL-1β, and IL-6 secretion with ELISA kits (R &D, Minneapolis, MN).

### Cell migration assay

Cell migration was measured using 24-well transwells with inserts containing 8-μm membrane filters (Corning Costar, Cambridge, MA). THP-1 cells treated with 320 nM of PMA for 24 h were seeded on 24-well plates (1 × 10^6^/well) and treated with various conditioned media collected from A549 cells or their derivatives for at least 24 h. The cells were replenished with the fresh medium containing 10% FBS and seeded on the lower chamber of transwells. Parental A549 cells resuspended in serum-free medium were added to the upper chamber (5 × 10^4^/well). The cells were cocultured for 6 h. Non-migratory cells on the upper surface of the membrane were removed with cotton rods. Migratory cells on the lower surface were fixed, stained with Giemsa solution, and photographed. Migratory cells were counted using the Image J software (National Institutes of Health, Bethesda, MD).

### Animal experiments

LL2 cells (5 × 10^5^) that had been transduced with recombinant retroviruses encoding mouse Oct4 or with control retroviruses were inoculated subcutaneously into the right flank of C57BL/6 mice. In addition, mice subcutaneously inoculated with LL2 cells (5 × 10^5^) at day 0 were treated intratumorally with 10 mg/kg of all-*trans* retinoic acid (ATRA) at days 10, 11, 13, 14, 25, 26, 28, and 29. All of the mice were monitored for tumor growth and survival. Tumor volumes were calculated as: length × width^2^ × 0.45. Mice were killed and regarded as deaths when tumors exceeded 2,000 mm^3^.

### Statistical analysis

Statistical significance between groups was determined with Student’s *t* test. Differences in tumor volume between two groups were compared by repeated-measures analysis of variance (ANOVA). Survival and recurrence-free curves were calculated by the Kaplan-Meier survival method and compared by the log-rank test. Correlations were measured using Pearson’s correlation coefficient (*r*). Any *p* value of < 0.05 is regarded as statistically significant.

## Results

### Expression of Oct4 in lung cancer cells induces monocyte differentiation into M2-like macrophages through M-CSF secretion

**Fig. 1:**
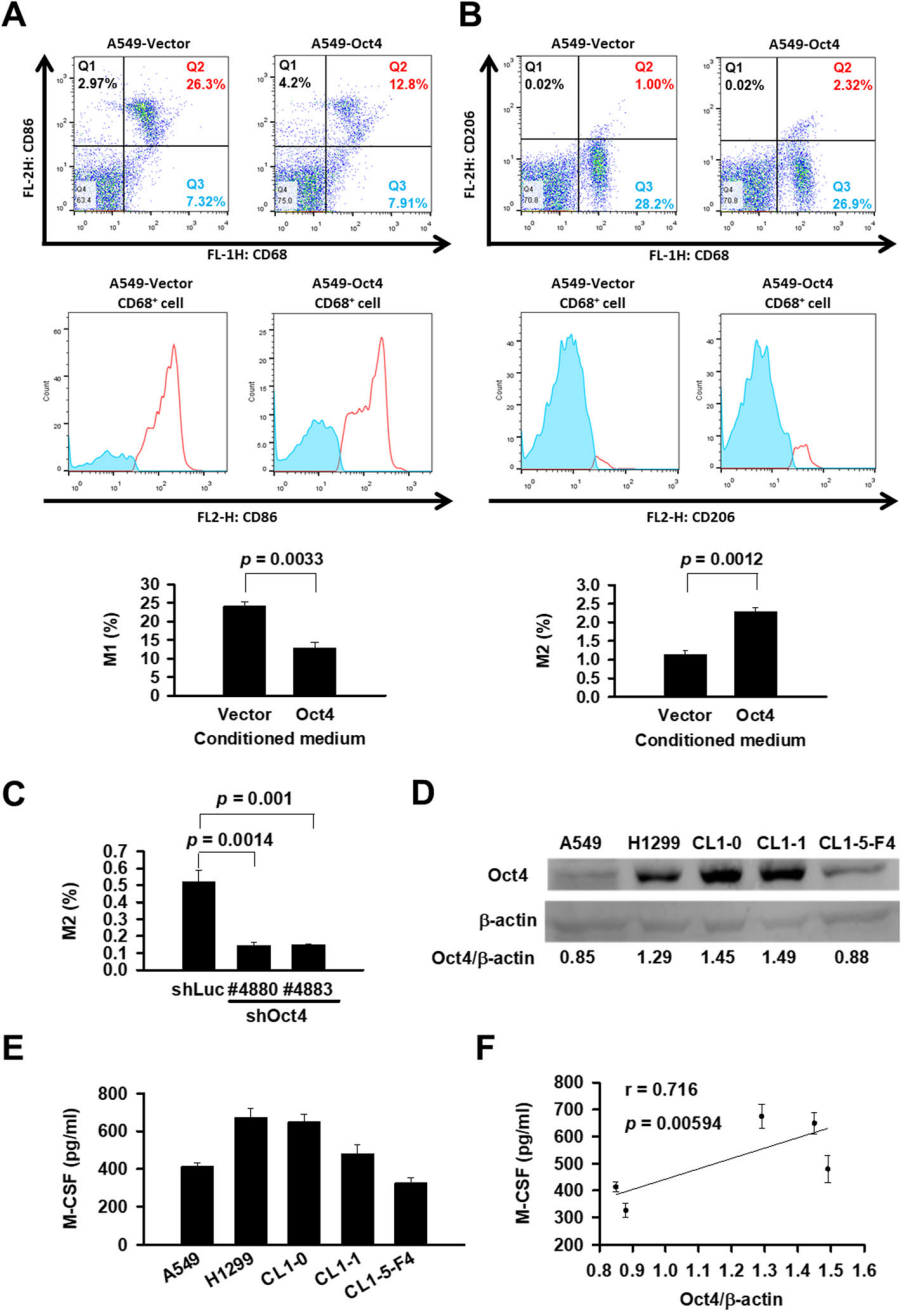
M2 macrophage polarization is associated with Oct4 expression in lung cancer. **a** and **b** Flow cytometric analysis of M1 and M2 macrophages differentiated from PMA-stimulated THP-1 cells cocultured with the conditioned medium obtained from A549-Oct4 or A549-vector cells. Cells were then stained with PE-conjugated mouse anti-human CD86 or CD206 antibody, followed by stained with FITC-conjugated mouse anti-human CD68 antibody before flow cytometric analysis. Cells were first gated to exclude debris and dead cells (FSC vs. SSC), and then gated to exclude cell doublets (FL2-A vs*.* FL2-H). Within the CD68^+^ cells, differential expression of the M1 macrophage marker CD86 and M2 macrophage marker CD206 is based on CD86hi and CD206hi expression, respectively. Representative dot plots (upper) and histograms (middle), as well as percentages of M1 and M2 macrophages (lower) are shown. **c** Percentage of M2 macrophages differentiated from PMA-stimulated THP-1 cells cocultured with the conditioned medium obtained from Oct4 knockdown (shOct4) or control (shLuc) A549 cells. **d** and **e** Oct4 expression (D) and M-CSF production (E) in various human lung cancer cells detected by immunoblot analysis and ELISA, respectively. Expression of β-actin served as the loading control. **f** Positive correlation of Oct4 and M-CSF levels detected in D and E, as determined by Pearson’s correlation coefficient. All data shown represent means ± SEM (*n* = 3 to 4)

**Fig. 2:**
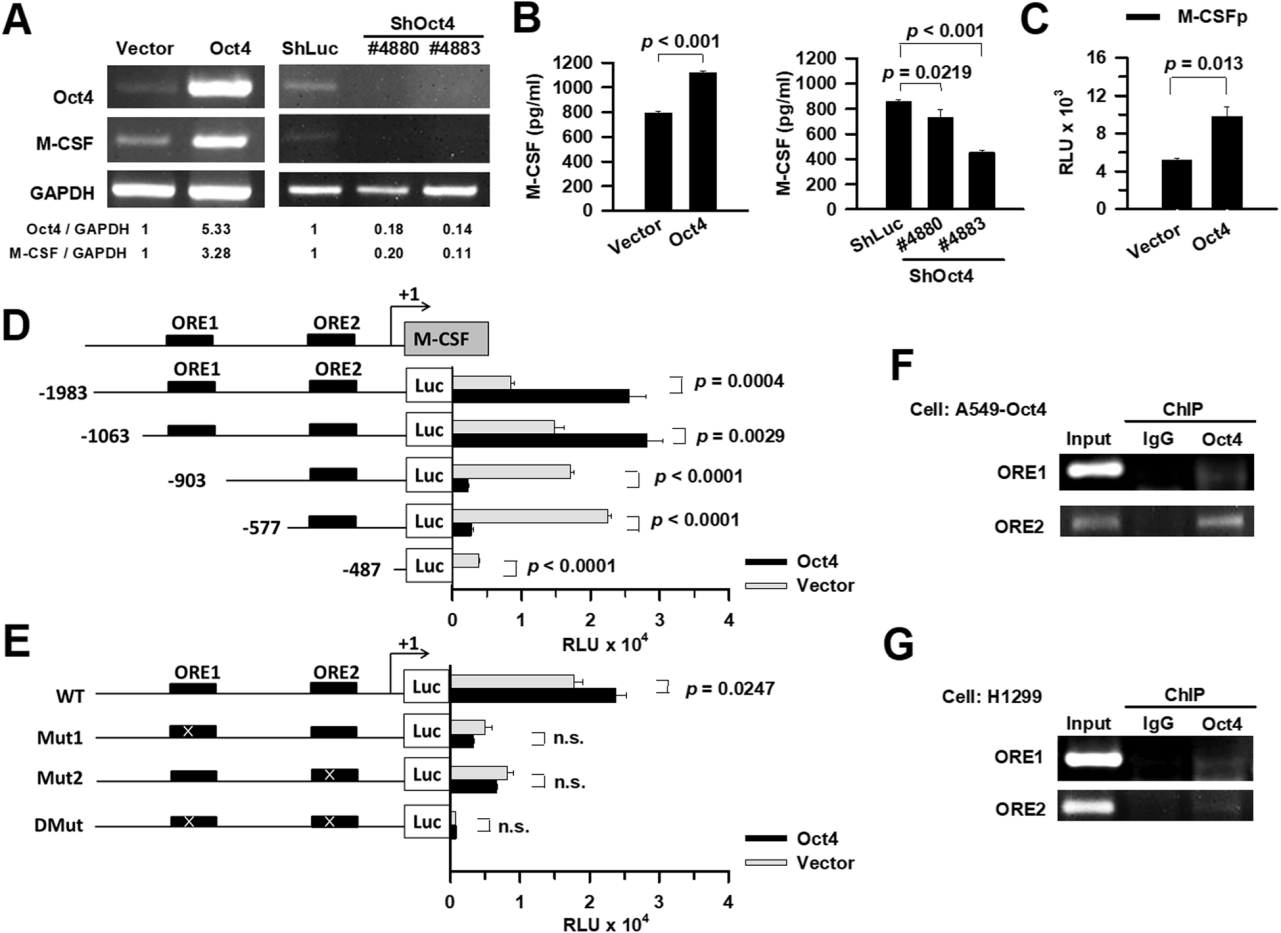
Oct4 directly binds and transactivates the *M-CSF* promoter in lung cancer cells. **a** and **b** Upregulation of M-CSF mRNA and protein levels in Oct4-overexpressing A549 cells (left panel) and downregulation of their levels in Oct4 knockdown A549 cells (right panel), as determined by RT-PCR (**a**) and ELISA (**b**). Expression of GAPDH served as the loading control. Values shown at the bottom of the ethidium bromide-stained gels are the ratios between the intensity of the bands corresponding to the indicated mRNA, as determined by densitometric analysis. The ratios of A549-vector cells are set as 1. **c** Transactivation of the *M-CSF* promoter by Oct4. **d** and **e** Schematic representation of a series of deletion constructs containing different lengths of the *M-CSF* promoter (**d**) and mutant constructs containing point mutations within the ORE1 and/or ORE2 sites (**e**). The black box indicates putative OREs. Numbering is relative to the translational start site at +1. Luciferase (Luc) reporter assays of various *M-CSF* promoter constructs. Two hundred ninety-three T cells were cotransfected with pSin-EF-Oct4-Pur (or control vector pSin-null) and a wild-type (**c**), deletion (**d**), or mutant (**e**) pFRL2-M-CSF construct. Cell lysates were harvested 48 h later, and their firefly and Renilla luciferase activities were determined using a dual-light luciferase reporter assay system. The ratio of firefly luciferase activity to *Renilla* luciferase activity was expressed as relative light units (RLU). **f** and **g** ChIP analysis showing direct binding of Oct4 to the ORE-containing region in the *M-CSF* promoter in A549-Oct (**f**) and H1299 (**g**) cells. Cross-linked chromatin was immunoprecipitated with anti-Oct4 or anti-IgG antibody combined with protein G agarose beads, followed by PCR amplification of the *M-CSF* promoter region that encompasses the ORE1 and ORE2 sites. Data shown represent means ± SEM (*n* = 3 to 4)

As human cancer cell lines are more closely resemble clinical human tumors than murine cancer cell lines, we employed human A549 lung cancer cells to study whether Oct4 expression in cancer cells contributed to macrophage polarization in vitro. The human monocytic leukemia THP-1 cells capable of differentiating into macrophage-like cells were cocultured with A549-Oct4 cells in the transwells, which could limit the macrophage-cancer cell interaction to occur only by diffusing factors. The percentages of cells stained positively for both CD68 and CD86 indicative of M1 macrophages were lower (Fig. 1a), whereas the percentages of CD68/CD206 double-positive cells indicative of M2 macrophages were higher (Fig. 1b), in THP-1 cells cocultured with the conditioned medium obtained from A549-Oct4 cells than those from vector control cells. To further validate the role of Oct4 in M2 macrophage polarization, we generated lentivirus-mediated stable Oct4-knockdown A549 cells and their vector control cells using two shRNA sequences targeting Oct4. The gene knockdown efficiency was over 80%, as determined by RT-PCR analysis (Fig. 2a, right). The percentages of M2 macrophages were reduced in THP-1 cells cocultured with the conditioned medium obtained from Oct4-knockdown A549 cells, as compared with those from the control cells (Fig. 1c). Taken together, these results suggest that Oct4-overexpressing lung cancer cells can induce monocytes to differentiate into M2 macrophages.

We next used human inflammation antibody arrays (ab134003, abcam) to identify the profile of inflammatory factors in the conditioned medium of A549 cells after transduction with lentiviral vectors encoding human Oct4 or with control lentiviral vectors for 72 h (Additional file 2: Figure S1A). Among 40 human inflammatory factors screened, 17 factors were detected in the conditioned medium of Oct4-overexpressing A549-Oct4 and vector control (A549-Vector) cells (Additional file 2: Figure S1B). The quantitative results show 1.36 ± 0.15-fold increases in M-CSF, 1.29 ± 0.01-fold increases in MCP-1, and 1.27 ± 0.04-fold increases in IL-6 levels in the conditioned medium of A549-Oct4 cells as compared with that of A549-Vector cells (Additional file 2: Figure S1C). We further examined the correlation between Oct4 expression and M-CSF production in various human lung cancer cell lines. H1299, CL1-0, and CL1-1 cell lines expressed higher levels of Oct4 (Fig. 1d) and concomitantly secreted higher amounts of M-CSF (Fig. 1e) than A549 and CL1-5-F4 cell lines. Notably, there was a positive correlation between the expression of Oct4 (Fig. 1d) and secretion of M-CSF (Fig. 1e) in these human lung cancer cells (Fig. 1f).

### Oct4 transactivates the *M-CSF* promoter

The levels of M-CSF mRNA (Fig. 2a, left) and protein (Fig. 2b, left) were higher in A549-Oct4 cells than in vector control cells. In contrast, knockdown of Oct4 expression dramatically reduced the expression levels of M-CSF mRNA (Fig. 2a, right) and protein (Fig. 2b, right) in A549 cells. As a transcription factor, Oct4 regulates downstream genes through its binding to the Oct4 ORE in embryonic stem cells [27]. We examined whether Oct4 could transactivate the *M-CSF* promoter. Luciferase reporter assay showed that Oct4 was capable of enhancing the promoter activity of M-CSF (Fig. 2c). To map the region in the *M-CSF* promoter required for transactivation by Oct4, the promoter region was examined by a deletion analysis. Deletions with the *M-CSF* promoter region between −1063 to −903 bp and −577 to −487 bp dramatically abolished its responsiveness to Oct4 in 293 T cells (Fig. 2d), suggesting that the ORE within these regions may be involved in Oct4-induced transactivation of the promoter. We used the PROMO computer software to predict transcription factor binding sites [28]. There are two potential OREs located on the *M-CSF* promoter. We designed mutant sequences of the ORE1 (ATGCAATT ➔ ACGCGATT) at −980 bp and the ORE2 (ATGCAAAT ➔ CTGAAGAT) at −530 bp, and examined whether these mutations abrogated Oct4 binding. Indeed, both mutations abolished Oct4-mediated transactivation, indicating that these OREs contributed to Oct4-induced transactivation of the *M-CSF* promoter (Fig. 2e). ChIP assay revealed that Oct4 bound to ORE1- and ORE2-containing regions within the *M-CSF* promoter in A549-Oct4 (Fig. 2f) and H1299 cells (Fig. 2g). Collectively, these results indicate that Oct4 can transactivate the *M-CSF* gene by binding to two regions between −980 bp and −530 bp of the promoter.

### M1 macrophages secrete TNF-α, IL-1β, and IL-6 to promote Oct4 expression in lung cancer cells

Inflammatory cells in the tumor microenvironment can affect the behavior of cancer cells [29]. Different types of macrophages not only affect cytokine expression but also interfere with surrounding cancer cells. Levels of TNF-α, IL-1β, and IL-6 secreted from M1 macrophages were all significantly higher than those secreted from M0 macrophages (Additional file 3: Figure S2A). Furthermore, A549 cells cocultured with M1 macrophages expressed higher levels of Oct4 than those cocultured with M0 microphages (Additional file 3: Figure S2B). Elevation of Oct4 expression was observed in A549 cells treated with TNF-α, IL-1β, or IL-6 (Additional file 3: Figure S2C). Moreover, such effects were more evident in cells treated with either TNF-α or IL-1β than in those treated with IL-6 at 10 ng/ml. Activation of the NF-κB pathway induces NSCLC initiating cells through regulating the EMT mechanism. Spheroid populations of A549 cells exhibit elevated expression of Oct4 [30]. We further confirmed that treatment with the NF-κB inhibitor JSH-23 (Sigma-Aldrich) inhibited Oct4 expression in A549 cells following treatment with TNF-α or IL-1β (Additional file 3: Figure S2D), suggesting that TAMs secret cytokines to upregulate Oct4 expression in lung cancer cells and subsequently promote differentiation of monocytes into M2 macrophages, leading to favoring tumor growth.

### M-CSF secreted by Oct4-overexpressing lung cancer cells contributes to macrophage-mediated promotion of cancer cell migration

**Fig. 3:**
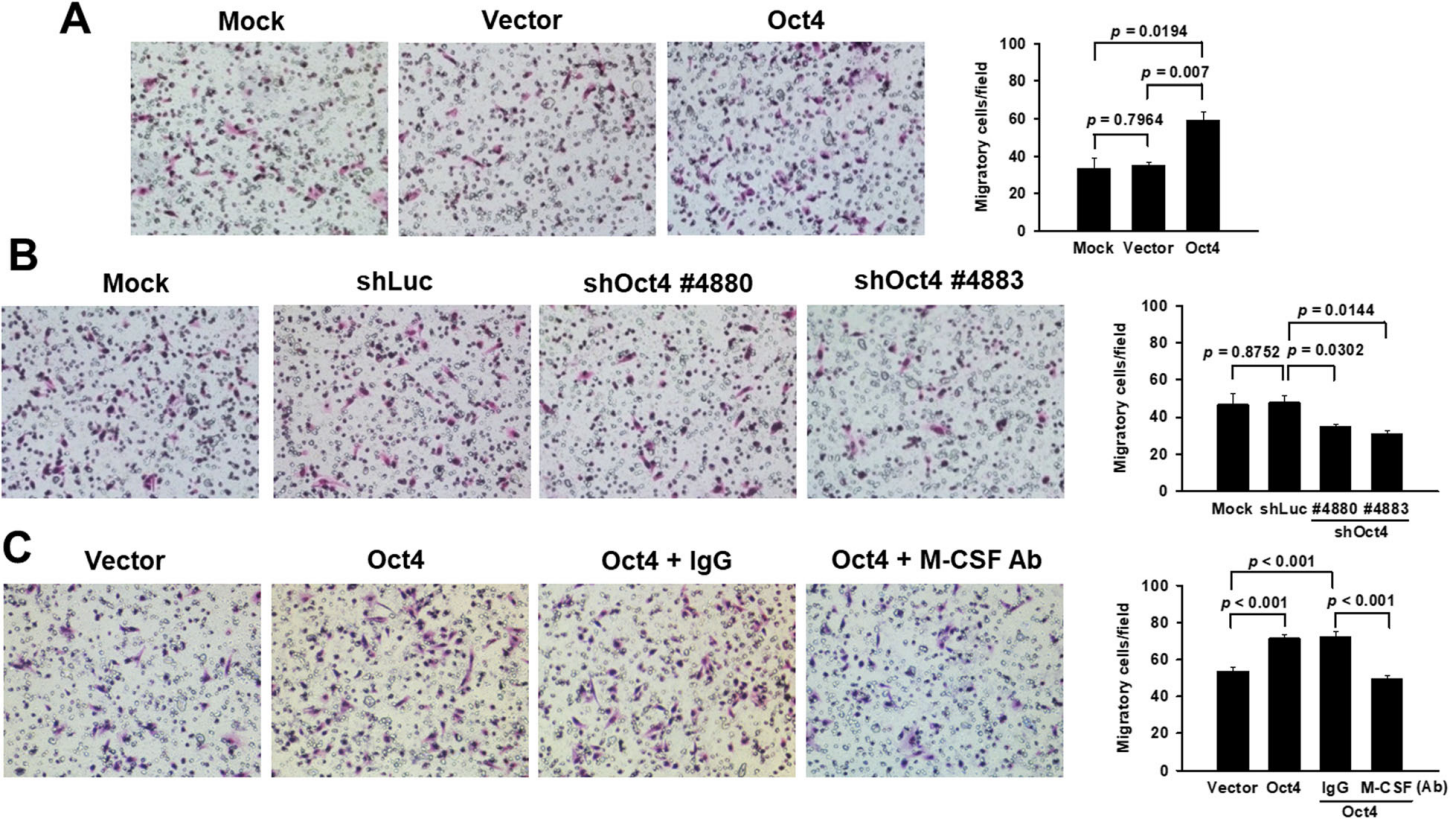
Macrophages stimulated by various tumor conditioned media affect the migratory ability of lung cancer cells. Transwell migration assay of A549 cells in response to THP-1 cells that had been treated with various conditioned media of A549 derivatives. A549 cells seeded in the upper chamber of transwells with inserts containing 8-μm membrane filters were cocultured with PMA-stimulated THP-1 cells, which had been treated with various conditioned media collected from A549-Oct4 cells (**a**), Oct4 knockdown A549 cells (**b**), or A549-Oct4 cells in the presence of anti-M-CSF antibody or control IgG (**c**), seeded in the lower chamber. After 6 h, A549 cells that migrated through the membrane to the lower surface were stained and quantified. Data shown represent means ± SEM (*n* = 3 in **a**, **b**; *n* = 10 to 12 in **c**). Data shown represent mean ± SEM. Results are representative of two independent experiments

Given that M2-like macrophages were induced by the conditioned medium of lung cancer cells, we investigated the impact of these macrophages on cancer cells. A549 cells were added into the upper chamber of the transwell, and THP-1-derived macrophages that had been stimulated by the conditioned medium collected from A549-Oct4 or vector control cells were added into the lower chamber of the transwell. Macrophages stimulated by the conditioned medium from A549-Oct4 cells significantly enhanced the migration of A549 cells compared with their two control counterparts (Fig. 3a). In contrast, macrophages stimulated by the conditioned medium from Oct4-knockdown A549 cells decreased cancer cell migration (Fig. 3b). Notably, the enhanced migration could be abrogated by the addition of anti-M-CSF antibody (Fig. 3c). Taken together, these results demonstrate that M-CSF produced by Oct4-overexpressing lung cancer cells stimulates macrophages to promote lung cancer cell migration.

### Overexpression of Oct4 in lung cancer increases M2 macrophages and enhances tumor metastasis in mice bearing syngeneic tumors

**Fig. 4:**
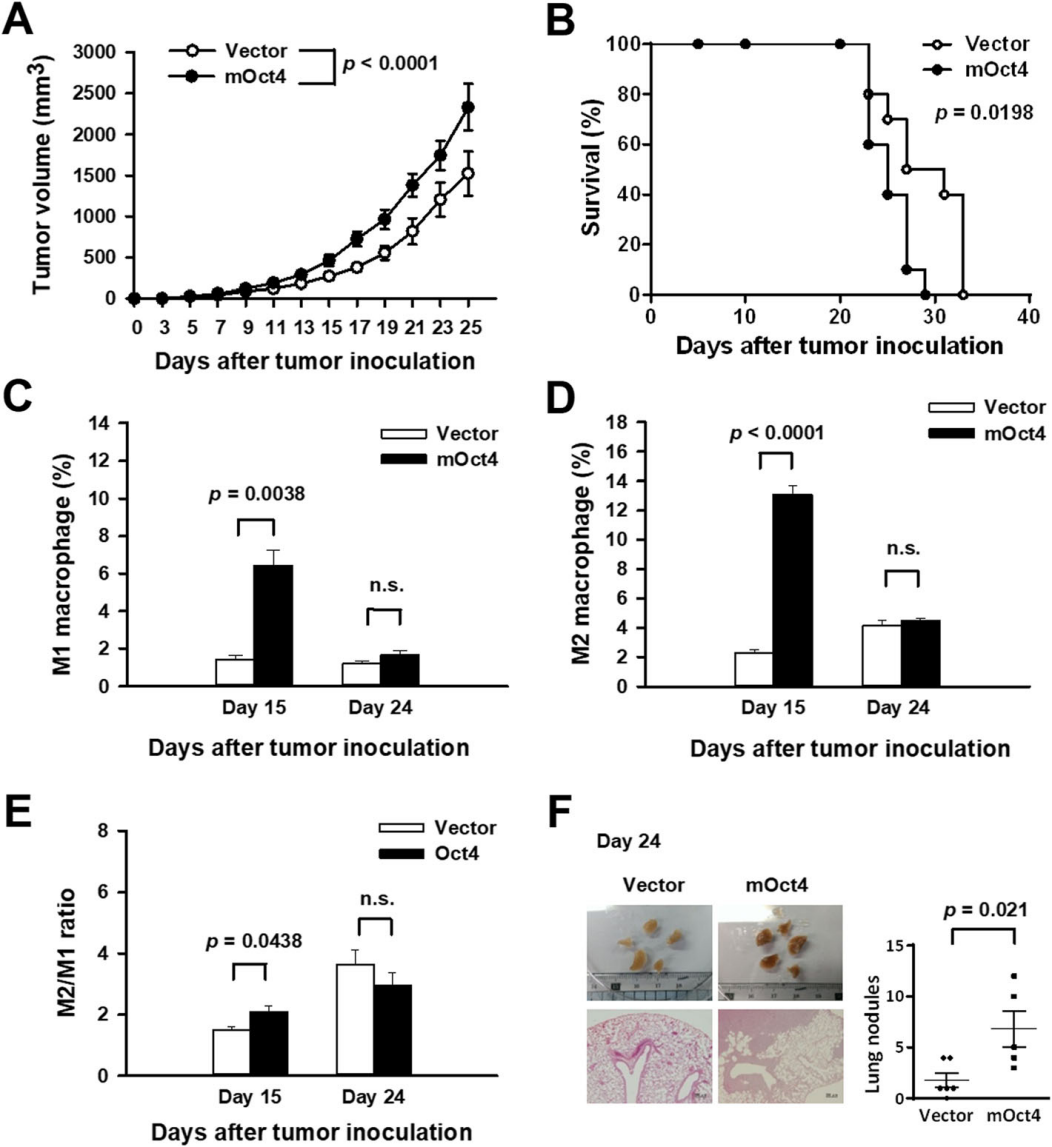
Overexpression of Oct4 in lung cancer promotes tumor growth, reduces survival, increases M2 macrophage infiltration, and enhances tumor metastasis in mice. **a** and **b** Tumor volumes (**a**) and survival curves (**b**) of C57BL/6 mice bearing LL2-Oct4 tumors (*n* = 10) or vector control tumors (*n* = 10). **c-e** Percentages of M1 (**c**) and M2 (**d**) macrophages and M2/M1 ratio (**e**) in LL2-Oct4 and vector control tumors at days 15 and 24 after tumor cell inoculation, as determined by flow cytometry for M1 macrophages positively stained for F4/80 and CD86 and for M2 macrophages positively stained for F4/80 and CD206. Data shown represent means ± SEM (*n* = 3 to 6). **f** Gross appearance of lungs (upper left panels), histology of tumor nodules in the lungs (lower left panels) (× 40 magnification, scale bar = 200 μm), and numbers of tumor nodules in individual mice with horizontal lines denoting means ± SEM (*n* = 5 to 6) (right panel) at day 24 after LL2 tumor cell inoculation. Results are representative of two independent experiments

Compared to xenograft models in immunodeficient mice, syngeneic tumor models in immunocompetent mice provide the appropriate tumor microenvironment and immunologic compatibility between tumor cells and the animal that is more closely representative of the mouse tumor and also more closely exemplifies the human and clinical physiology [31]. Thus, we used LL2 lung tumors grown in syngeneic C57BL/6 mice to investigate whether overexpression of Oct4 in lung cancer cells impacted tumor growth and infiltration of M1/M2 macrophages in the tumors. C57BL/6 mice bearing subcutaneous LL2-Oct4 tumors had larger tumor volumes (Fig. 4a) and shorter survival time (Fig. 4b) compared with those bearing control LL2 tumors. We further analyzed macrophage population in tumors. At day 15, the percentages of both M1 and M2 macrophages were higher in LL2-Oct4 tumors than in control tumors (Fig. 4c, d, and Additional file 4: Figure S3). However, percentage of either M1 or M2 macrophages was similar between LL2-Oct4 and control tumors at day 24. Notably, the ratio of M2/M1 macrophages in LL2-Oct4 tumors was higher than that in control tumors at day 15 (Fig. 4e). In terms of tumor metastasis detected at day 24, numbers of metastatic tumor nodules in the lung are significantly greater in mice bearing LL2-Oct4 tumors compared with those in mice bearing control tumors (Fig. 4f).

### Treatment with ATRA decreases tumor metastasis and expression of Oct4, M-CSF, and CD206 in mice

**Fig. 5:**
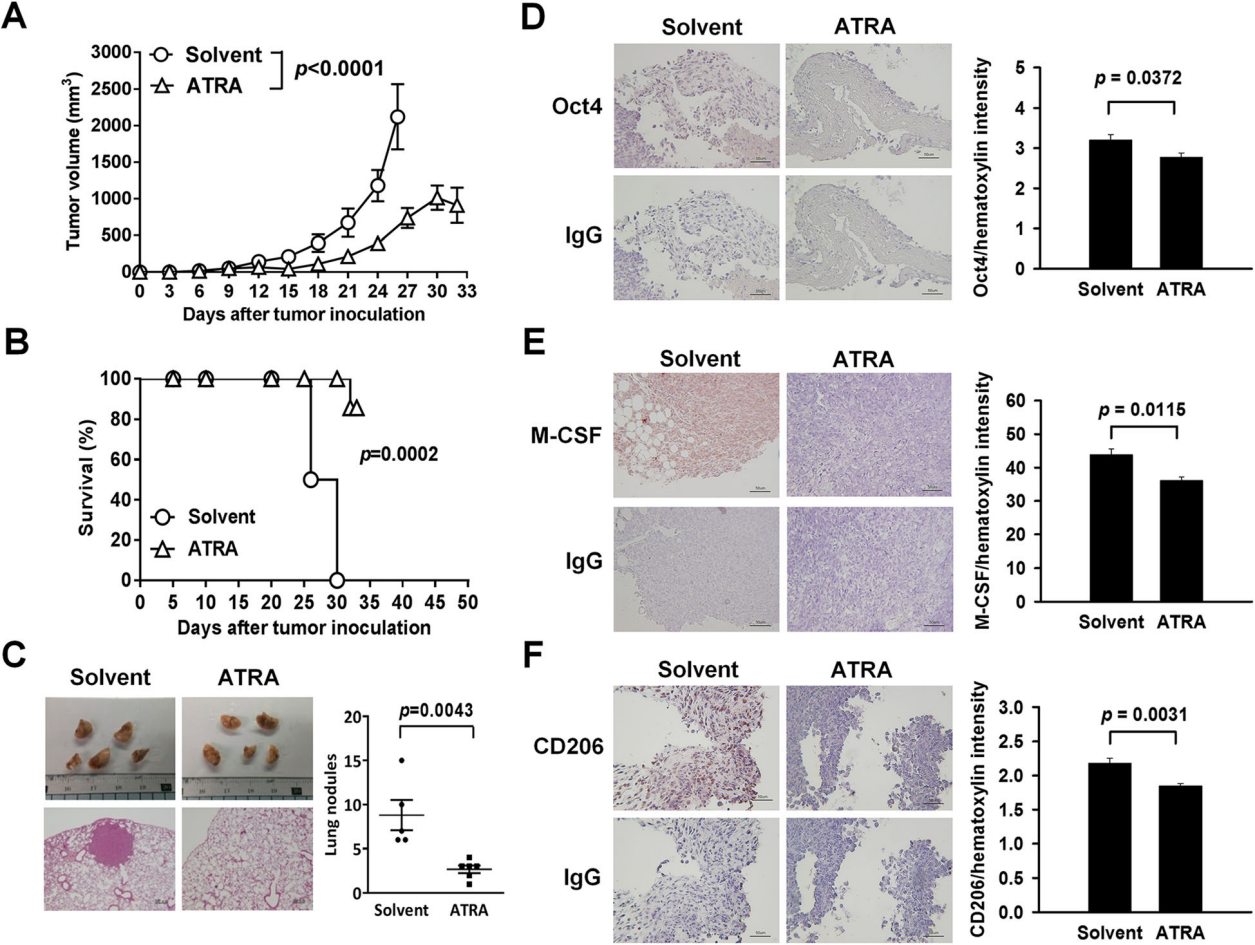
Treatment with ATRA retards tumor growth, prolongs survival, decreases tumor metastasis, and reduces M2 macrophage infiltration in LL2 tumor-bearing mice. **a** and **b** Tumor volume (**a**) and survival curves (**b**) of tumor-bearing mice (*n* = 6 in the solvent group; *n* = 7 in the ATRA group;). **c** Gross appearance of lungs (upper left panels), histology of tumor nodules in the lungs (lower left panels) (× 40 magnification, scale bar = 200 μm), and numbers of tumor nodules in individual mice with horizontal lines denoting means ± SEM (*n* = 5 to 6) (right panel) at day 35 after LL2 tumor cell inoculation. C57BL/6 mice were subcutaneously inoculated with LL2 cells (5 × 10^5^) at day 0 and treated intratumorally with ATRA (10 mg/kg) at days 10, 11, 13, 14, 25, 26, 28, and 29. **d-f** Immunohistochemical detection of Oct4, M-CSF, and CD206 in tumor tissues of mice after treatment with ATRA or solvent. Immunohistochemical staining of lung tissues (left panels) (× 200 magnification) and quantification of immunoreactive intensities of Oct4 (**d**), M-CSF (**e**), and CD206 (**f**) in AEC-positive areas (right panels) at day 15 after tumor cell inoculation (*n* = 6 in the solvent group; *n* = 4 to 5 in the ATRA group). Negative control slides stained with isotype control IgG. C57BL/6 mice were subcutaneously inoculated with LL2 cells (5 × 10^5^) at day 0 and treated intratumorally with ATRA (10 mg/kg) at days 10, 11, 13, 14. Results are representative of two independent experiments. Scale bar, 50 μm

ATRA exerts a potent activity in stem cell differentiation through several retinoid acid-responsive elements on the promoter-enhancer regions of its target genes, including Oct4 [32]. Given that Oct4 is a major player of Oct4/M-CSF axis in M2 macrophages associated with tumor progression, we used ATRA to inhibit Oct4 expression and analyzed M2 macrophages in the LL2 lung tumor model. Treatment with ATRA (10 mg/kg) significantly retarded tumor growth (Fig. 5a) and prolonged the survival time (Fig. 5b) in tumor-bearing mice. When the tumor size exceeded 2000 mm^3^, mice were euthanized and regarded as deaths. Furthermore, numbers of lung tumor nodules were decreased in ATRA-treated mice (Fig. 5c). Moreover, expression of Oct4 (Fig. 5d), M-CSF (Fig. 5e), and CD206 (Fig. 5f) was decreased in the tumors of ATRA-treated mice. Collectively, these results indicate that ATRA inhibits tumor growth and metastasis, as well as decreases M2 macrophages and M-CSF levels.

### Expression levels of Oct4, M-CSF, and CD206 are elevated in clinical lung tumor specimens and positively correlated with each other

**Fig. 6:**
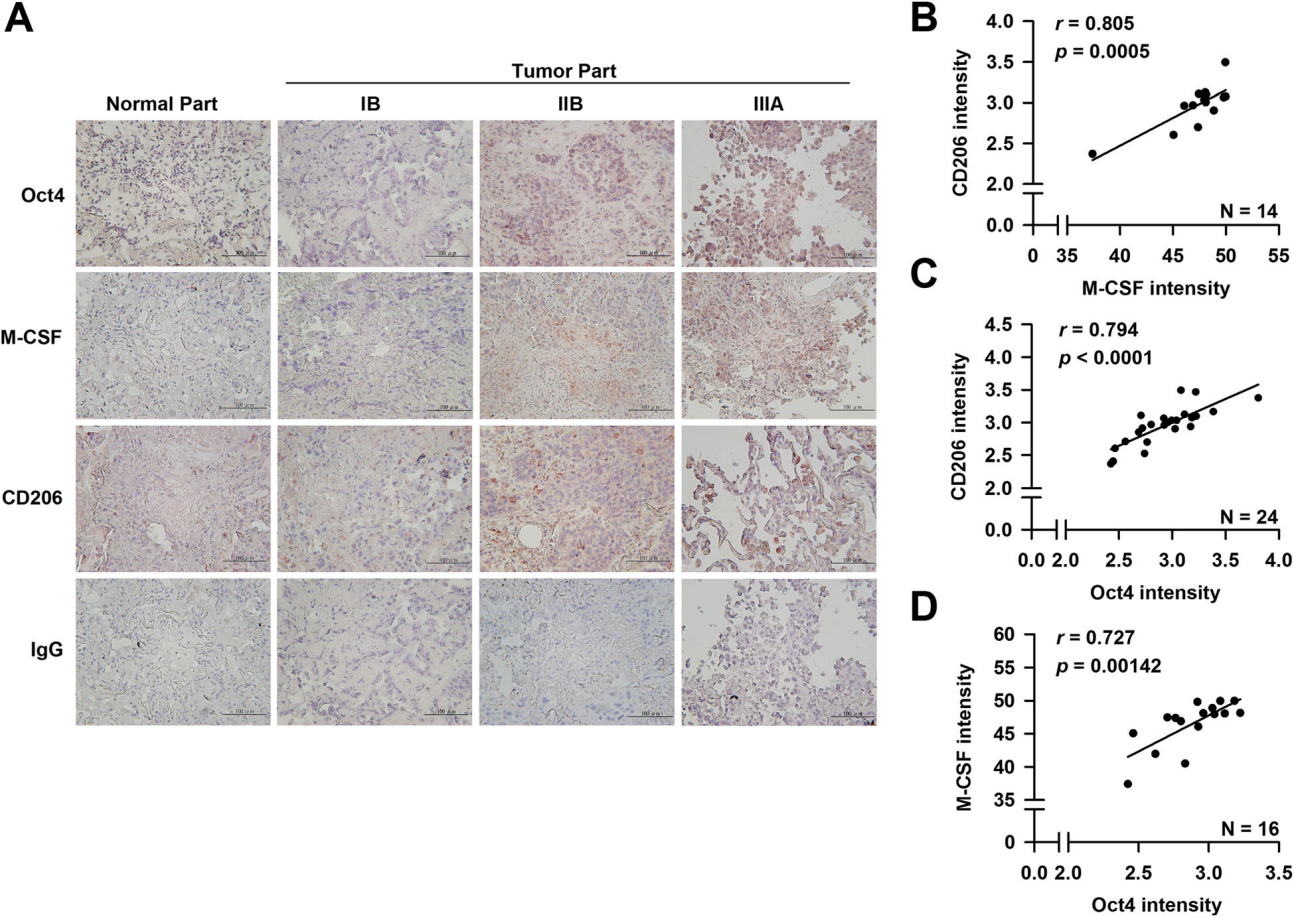
Expression of Oct4, M-CSF, and CD206 is positively correlated with each other in clinical lung tumor tissues. **a** Representative immunohistochemical staining of Oct4, M-CSF, and CD206 (M2 macrophage marker) in tumor tissues of patients with stages IB, IIB, and IIIA lung cancer and in normal tissues (× 200 magnification, scale bar = 100 μm). Negative control slides stained with isotype control IgG. **b-d** Immunoreactive intensities of Oct4, M-CSF, and CD206 were quantified, and the correlations between the levels of M-CSF and CD206 (*n* = 15) (**b**), Oct4 and CD206 (*n* = 22) (**c**), and Oct4 and M-CSF (*n* = 17) (**d**) were determined by Pearson's correlation coefficient. Scale bar, 100 μm

To determine whether the expression profiles of Oct4, M-CSF, and CD206 in lung cancer have clinical relevance, we examined their expression and correlation in clinical specimens of 22 patients with stages IB, IIB, and IIIA lung cancers. As shown in Fig. 6a, tumor tissues, in particular invasive tumors (stages IIB and IIIA), expressed elevated levels of Oct4, M-CSF, and CD206 compared with normal tissues. Furthermore, CD206-positive cells indicative of M2 macrophages were evident in lung tumor tissues (Fig. 6a). Notably, there were positive correlations between the expression levels of M-CSF and CD206 (Fig. 6b), Oct4 and CD206 (Fig. 6c), as well as Oct4 and M-CSF (Fig. 6d). These results from clinical samples indicate that Oct4, M-CSF, and CD206 are positively inter-correlated.

### The expression status of Oct4, M-CSF, and CD206 in combination is correlated with prognosis of patients with lung cancer

**Fig. 7:**
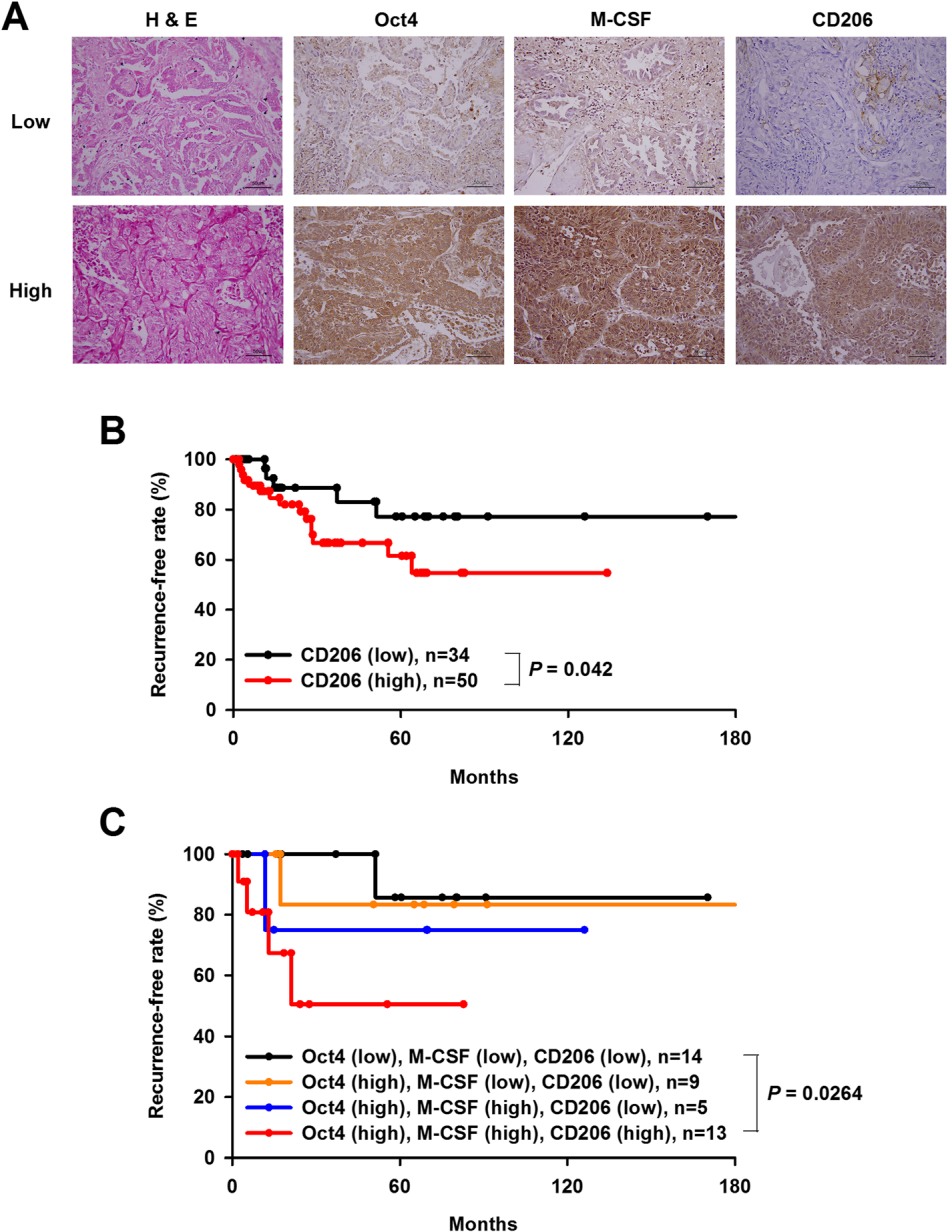
High levels of Oct4, M-CSF, and CD206 expression are correlated with tumor recurrence in patients with stages IB, IIB, and IIIA lung cancer. **a** Immunoreactivity of Oct4, M-CSF, and CD206 was categorized as low and high in tumor samples of patients with lung cancer. **b** and **c** Kaplan-Meier curves of recurrence-free rate in patients with high or low expression of CD206 (**b**), as well as with combinations of different levels of Oct4, M-CSF, and CD206 expression (**c**). Differences in recurrence-free intervals were analyzed by the log-rank test. Scale bar, 50 μm

To evaluate the roles of Oct4, M-CSF, and M2 macrophages in tumor recurrence, another cohort with 84 lung cancer patients was analyzed. Immunoreactive intensity for Oct4, M-CSF, and CD206 was individually categorized as low or high (Fig. 7a). Kaplan-Meier analysis shows that patients with CD206 high-expressing tumors had significantly lower recurrence-free rates than those with CD206 low-expressing tumors (Log-rank test, *p* = 0.042) (Fig. 7b). Furthermore, when the three determinants were analyzed in combination, patients with high expression levels of Oct4, M-CSF, and CD206 experienced a worse outcome of recurrence-free survival, compared with those with low expression levels of the three molecules (Log-rank test, *p* = 0.0264) (Fig. 7c). Therefore, the expression of Oct4, M-CSF, and CD206 in combination is positively correlated with tumor recurrence in patients with lung cancer.

## Discussion

Chronic inflammation has been recognized as an important contributor in the development of a variety of malignancies. Macrophages are key immune cells in chronic inflammation. TAMs have been shown to be continuously replenished in vivo, mostly by circulating inflammatory Ly6C^hi^ monocytes, which are cells that foster tumor progression [33]. TAMs are associated with decreased survival in patients with colorectal cancer [34], melanoma [35], breast cancer [36], and bladder cancer [37]. In the present study, we show that M1 macrophages can induce Oct4 expression in lung cancer cells, which reinforces the interaction between monocyte lineage and cancer cells and highlights the role of macrophages in tumor initiation. Production of TNF-α, IL-1β, and IL-6 is accompanied with M1 macrophage differentiation, which may support antitumor immune responses. However, both TNF-α and IL-1β also upregulate Oct4 expression in cancer cells, which may counterbalance the antitumor activity of M1 macrophages. Oct4 also transactivates M-CSF gene expression in lung cancer cells. Furthermore, TAMs undergo M2 polarization in part through M-CSF stimulation to inhibit antitumor immune responses and lead to tumor recurrence.

M-CSF, an important factor for macrophage differentiation and survival, can induce M2 macrophage polarization [38]. Cancer stem-like cells can generate M2-like immunoregulatory myeloid cells from CD14^+^ monocytes by producing a variety of proinflammatory cytokines, including M-CSF [39]. Activation of M-CSF pathways in cancer cells is correlated with the number of tumor-associated M-CSF receptor-positive M2 macrophages in patients with lung cancer [39]. M-CSF expressed by glioblastoma cells plays a role in resistance to the anticancer drug 5-FU [40]. M-CSF derived from glioblastoma also promotes angiogenesis by microglial release of insulin-like growth factor-binding protein 1 [41]. Serum M-CSF levels in colorectal cancer patients have been shown to correlate with lymph node metastasis and poor prognosis [42]. In the present study, our data support the notion that M-CSF secreted by Oct4-overexpressing lung cancer cells, which can be upregulated, in part, by TNF-α and IL-1β secreted from M1 macrophages, promotes Oct4-mediated M2 macrophage polarization and thereby increases tumor progression.

Bidirectional communication between cells and their microenvironment is critical for both normal tissue homeostasis and tumor growth. Oct4, which controls self-renewal ability and pluripotency in embryonic stem cells, is highly expressed in various types of cancers and plays pivotal roles in tumorigenesis. It is therefore of importance to unravel how TAMs impact Oct4 expression in cancer cells. Activation of NF-κB has been shown to promote lung cancer metastasis [43]. TNF-α present in the tumor environment can induce cancer cells to display cancer stem-like phenotypes [44]. In the present study, we found that treatment with TNF-α or IL-1β increases Oct4 expression in lung cancer cells through NF-κB. These results suggest that increases in tumor-initiating cells may be associated with the inflammatory environment. Our results showing upregulation of Oct4 in lung cancer cells either by coculture with macrophages or with TNF-α or IL-1β provide supports for the acquired cancer stem cell hypothesis [45]. We have previously reported that the treatment of bladder cancer cells with the chemotherapeutic drug cisplatin induces Oct4 expression, leading to the acquisition of cancer stem cell phenotypes and resistance to cisplatin [16].

Despite the interesting results obtained from the present work, there are certain limitations that should be highlighted and discussed. TAMs originate as blood monocytes recruited from the tumor vasculature by tumor-derived signals, such as M-CSF, MCP-1, IL-4, and VEGF [46]. Furthermore, M-CSF, IL-4, IL10, and TGF-β secreted by tumor cells can trigger the M2 program [47]. In addition to M-CSF, among the inflammatory factors analyzed in our study, MCP-1 and IL-6 were increased, whereas eotaxin-2 and ICAM-1 were decreased in lung cancer cells overexpressing Oct4. These results suggest that MCP-1, IL-6, eotaxin-2, and ICAM-1 might also be regulated by Oct4 through direct or indirect mechanisms. ICAM-1 can suppress tumor metastasis by inhibiting M2 macrophage polarization through blockade of efferocytosis [48]. Nevertheless, MCP-1 and IL-6 have been reported to play important roles in the control of malignant pleural effusion and survival in mice [49] and patients with primary lung adenocarcinoma [50]. Tumor-derived MCP-1 can elicit effector T cell chemotaxis [51]. Taken together, these studies suggest that Oct4 may be involved in inducing malignant pleural effusion. Nevertheless, the underlying mechanism needs further investigation.

Targeting tumor-infiltrating macrophages decreases tumor-initiating cells, relieves immunosuppression, and improves chemotherapeutic responses [52]. In the present study, we found a positive correlation between expression of Oct4, M-CSF, and M2 macrophages in human lung cancer specimens. We have previously shown that inhibition of Oct4 expression by ATRA in bladder cancer cells renders cells less susceptible to cytolytic effects induced by an oncolytic adenovirus carrying the Oct4 response element (ORE) [53]. We have also demonstrated that inhibition of Oct4 expression by ATRA improves the sensitivity of cancer cells to cisplatin [16]. In animal work, we have used 10 mg/kg of ATRA in a bladder cancer study [16]. Our results reveal that combination treatment with cisplatin and ATRA is superior to cisplatin alone in suppressing bladder tumor growth in vivo. We then adopted this dose for LL2 tumor experiments. In our animal experiments, the mice suffered no significant weight loss after ATRA treatment. However, we did not examine other side effects or toxicity. In fact, acute renal failure during ATRA treatment in patients with acute promyelocytic leukemia has been reported [54]. Thus, kidney functions of the treated mice deserve further analysis. In an animal study, when mice were intraperitoneally treated with ATRA (35 mg/kg) 16 h before being injected with ConA, significantly less ALT activity was observed in the ATRA-treated mice [55]. These results suggest that ATRA at doses as high as 35 mg/kg is generally well tolerated in mice.

Our results showed that LL2 tumor-bearing C57BL/6 mice treated with ATRA had reduced tumor growth rate, better survival rate, concomitantly with decreased M2 macrophage infiltration, and lung metastasis. ATRA can enhance the cytotoxicity of low-dose cisplatin and 5-fluorouracil against CD44^+^ cancer stem cells [56]. ATRA and genistein can synergistically inhibit the metastatic potential of human lung adenocarcinoma cells [57]. Taken together, combination of ATRA with other current anticancer agents may be a promising strategy for lung cancer treatment. Given the important role of Oct4 in tumorigenesis and maintenance of cancer stem cell properties, Oct4-expressing cells may represent a specific subset of target cells for effective cancer therapy.

## Conclusions

In conclusion, our results from lung cancer patients, animal models, and in vitro studies indicate that M-CSF-mediated M2-polarized TAMs promote migration of cancer cells, suggesting that M2 macrophages are triggered by Oct4- overexpressing cancer cells and M-CSF may be a therapeutic target for lung cancer. Furthermore, inhibition of Oct4 expression by ATRA may be a potential therapeutic strategy of lung cancer.

## Supplementary information

**Additional file 1: Table S1.** Clinicopathological parameter of the present study population.

**Additional file 2: Figure S1.** Inflammatory factors secreted by Oct4-expressing cancer cells.

**Additional file 3: Figure S2.** M1 cytokines enhance Oct4 expression in A549 cells.

**Additional file 4: Figure S3.** Flow cytometric analysis of M1 and M2 macrophage in the tumors at day 15 in mice after inoculation of LL2-Oct4 or control LL2 tumor cells.

## Data Availability

The datasets used and/or analyzed during the current study are available from the corresponding author on reasonable request.

## Abbreviations

ATRA: All-*trans* retinoic acid
ChIP: Chromatin immunoprecipitation
CSC: Cancer stem cell
ELISA: Enzyme-linked immunosorbent assay
IHC: Immunohistochemistry
IL-1β: Interleukin-1β
LL2: Lewis lung carcinoma 2
MCP-1: Monocyte chemoattractant protein-1
M-CSF: Colony-stimulating factor
MIP-1α: Macrophage inflammatory protein-1α
NSCLC: Non-small cell lung cancer
Oct-4: Octamer-binding transcription factor 4
PCR: Polymerase chain reaction
PMA: Phorbol 12-myristate 13-acetate
RT-PCR: Reverse transcription-PCR
SEM: Standard error of the mean
TAMs: Tumor-associated macrophages
TNF-α: Tumor necrosis factor-α
