# Supplementary material for: Oct4 promotes M2 macrophage polarization through upregulation of macrophage colony-stimulating factor in lung cancer
Source: J Hematol Oncol. 2020 Jun 1;13:62. doi: 10.1186/s13045-020-00887-1 (PMC7268452; doi:10.1186/s13045-020-00887-1)
Supplement: Supplementary file 1 — Additional file 1: Table S1. Clinicopathological parameter of the present study population. [file 13045_2020_887_MOESM1_ESM.docx]

**Additional file 1: Supplementary Table**

Table S1. Clinicopathological parameters of the present study population.

Parameter *n* = 84

Median age (range), years 75 (49-90)

Sex

male 50

female 34

Tumor pathological stage

I 36

II 21

III 27

Histology subtypes

Adenocarcinoma 68

Squamous cell carcinoma 14

Adenosquamous carcinoma 1

Atypical carcinoid tumor 1

Lymph node involvement

negative 35

positive 49

Operative method

wedge resection 1

lobectomy 77

pneumonectomy 6

Oct4 expression (intensity)

Low 46

High 38

M-CSF expression (intensity)

Low 58

High 26

CD206 expression (intensity)

Low 50

High 34
