# Supplementary material for: Oct4 promotes M2 macrophage polarization through upregulation of macrophage colony-stimulating factor in lung cancer
Source: J Hematol Oncol. 2020 Jun 1;13:62. doi: 10.1186/s13045-020-00887-1 (PMC7268452; doi:10.1186/s13045-020-00887-1)
Supplement: Supplementary file 2 — Additional file 2: Figure S1. Inflammatory factors secreted by Oct4-expressing cancer cells. [file 13045_2020_887_MOESM2_ESM.docx]

**Additional file 2: Supplementary Figure**

**Supplementary Figure S1**


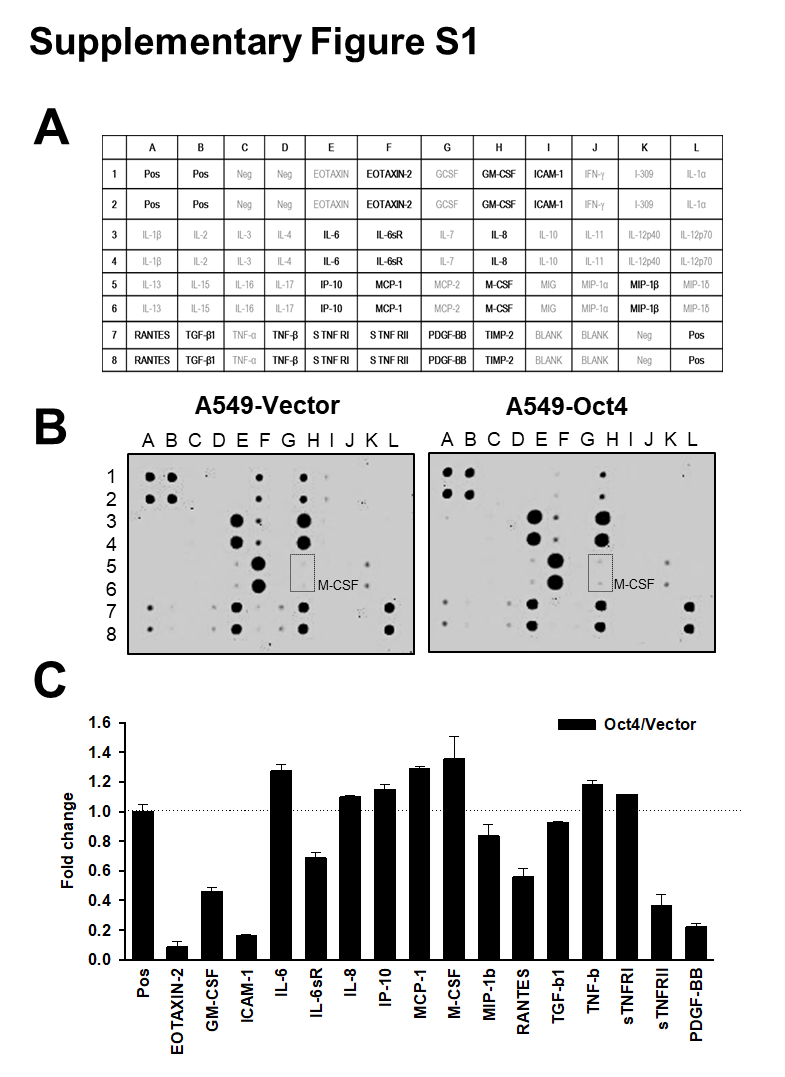


**Supplementary Figure S1.** Inflammatory factors secreted by Oct4-expressing cancer cells. The levels of 40 different inflammatory factors in the conditioned medium of A549 cells which transduced Oct4 or vector were determined with a human inflammation antibody array. **A,** The scheme of spots of various capture antibodies specific to inflammatory factors. POS and NEG denote positive and negative control spots, respectively. **B,** The dot plots of membrane. **C,** The semiquantitative analysis results of the dot plots of membranes. The levels of different factors were quantified using ImageJ software. Relative levels of inflammatory factors were determined using densitometry on the array. Each column represents mean ± SEM, n = 2.
