# Supplementary material for: Oct4 promotes M2 macrophage polarization through upregulation of macrophage colony-stimulating factor in lung cancer
Source: J Hematol Oncol. 2020 Jun 1;13:62. doi: 10.1186/s13045-020-00887-1 (PMC7268452; doi:10.1186/s13045-020-00887-1)
Supplement: Supplementary file 3 — Additional file 3: Figure S2. M1 cytokines enhance Oct4 expression in A549 cells. [file 13045_2020_887_MOESM3_ESM.docx]

**Additional file 3: Supplementary Figure**

**Supplementary Figure S2**


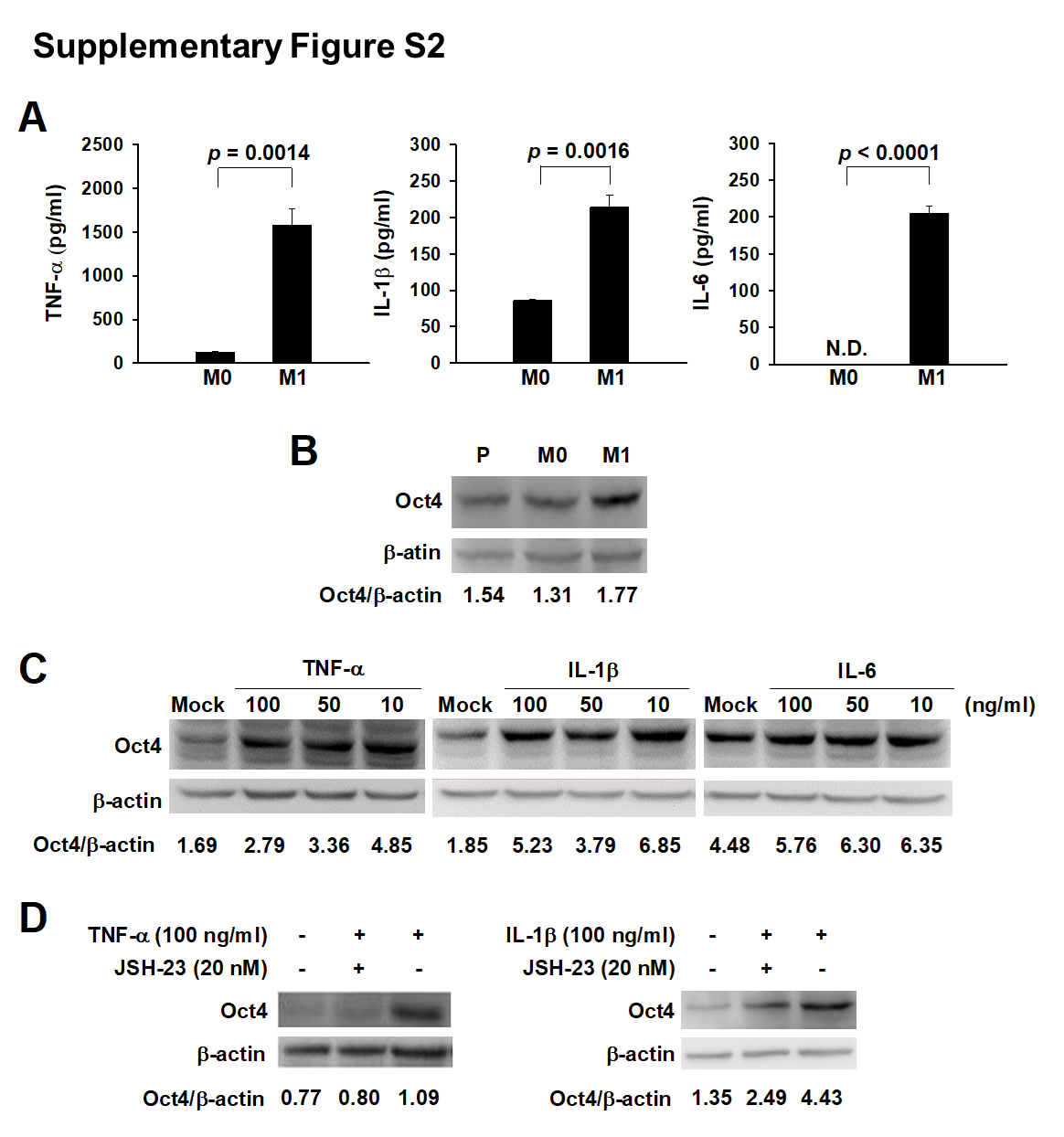


**Supplementary Figure S2.** M1 cytokines enhance Oct4 expression in A549 cells. **A,** M1-polarized THP-1 macrophages secreted significantly higher levels of TNF-α, IL-6 and IL-1β than did control cells. THP-1 cells were treated with 320 nM of PMA for 6 h and then cultured for an additional 16 h with either PMA only to generate M0 cells or PMA plus lipopolysaccharide (100 ng/ml)/interferon-γ (20 ng/ml) to generate M1-polarized cells. Secretion of TNF-α, IL-1β, and IL-6 by M0 and M1 macrophages were detected by ELISA. **B,** A549 cells were coincubated with M0, M1, or parental (P) THP-1 cells for 24 h. **C and D,** A549 cell were treated with TNF-α, IL-1β, IL-6 (**C**) and NF-κB inhibitors (**D**) for 24 h. Oct4 expression in A549 cells in **B-D** were analyzed by immunoblotting. Values shown at the bottom of the blots are the ratios between the intensity of the bands corresponding to Oct4 and those corresponding to β-actin, as determined by densitometric analysis.
