# Supplementary material for: Oct4 promotes M2 macrophage polarization through upregulation of macrophage colony-stimulating factor in lung cancer
Source: J Hematol Oncol. 2020 Jun 1;13:62. doi: 10.1186/s13045-020-00887-1 (PMC7268452; doi:10.1186/s13045-020-00887-1)
Supplement: Supplementary file 4 — Additional file 4: Figure S3. Flow cytometric analysis of M1 and M2 macrophage in the tumors at day 15 in mice after inoculation of LL2-Oct4 or control LL2 tumor cells. [file 13045_2020_887_MOESM4_ESM.docx]

**Additional file 4: Supplementary Figure**

**Supplementary Figure S3**

**
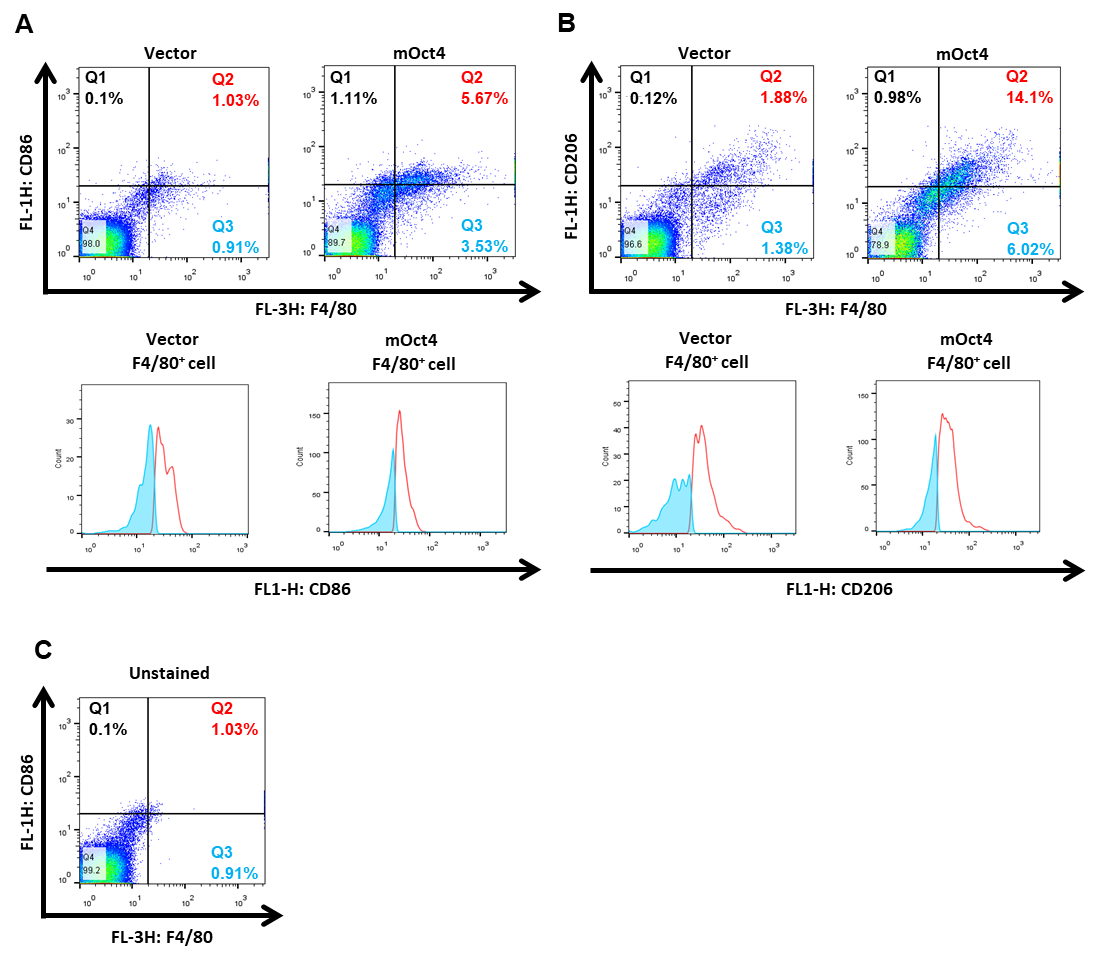
**

**Supplementary Figure S4.** Analysis of M1 and M2 macrophages in the mouse tumors at day 15 after inoculation of LL2-Oct4 or control LL2 tumor cells. The isolated cells were stained with PE-Cy7-conjugated rat anti-mouse F4/80, FITC-conjugated rat anti-mouse CD86, or FITC-conjugated rat anti-mouse CD206 antibody. Cells were first gated to exclude debris and dead cells (FSC vs. SSC), and then gated to exclude cell doublets (FL2-A vs. FL2-H). Within the CD68^+^ cells, differential expression of the M1 macrophage marker CD86 and M2 macrophage marker CD206 is based on CD86hi and CD206hi expression, respectively. Representative dot plots (upper) and histograms (lower) are shown. **C,** unstained cells.
